# Empirical Evidence for the Effect of Airline Travel on Inter-Regional Influenza Spread in the United States

**DOI:** 10.1371/journal.pmed.0030401

**Published:** 2006-09-12

**Authors:** John S Brownstein, Cecily J Wolfe, Kenneth D Mandl

**Affiliations:** 1 Children's Hospital Informatics Program at the Harvard–MIT Division of Health Sciences and Technology, Boston, Massachusetts, United States of America; 2 Division of Emergency Medicine, Children's Hospital Boston, Boston, Massachusetts, United States of America; 3 Department of Pediatrics, Harvard Medical School, Boston, Massachusetts, United States of America; 4 Hawaii Institute of Geophysics and Planetology, University of Hawaii at Manoa, Honolulu, Hawaii, United States of America; Yale University, United States of America

## Abstract

**Background:**

The influence of air travel on influenza spread has been the subject of numerous investigations using simulation, but very little empirical evidence has been provided. Understanding the role of airline travel in large-scale influenza spread is especially important given the mounting threat of an influenza pandemic. Several recent simulation studies have concluded that air travel restrictions may not have a significant impact on the course of a pandemic. Here, we assess, with empirical data, the role of airline volume on the yearly inter-regional spread of influenza in the United States.

**Methods and Findings:**

We measured rate of inter-regional spread and timing of influenza in the United States for nine seasons, from 1996 to 2005 using weekly influenza and pneumonia mortality from the Centers for Disease Control and Prevention. Seasonality was characterized by band-pass filtering. We found that domestic airline travel volume in November (mostly surrounding the Thanksgiving holiday) predicts the rate of influenza spread (*r* ^2^ = 0.60; *p =* 0.014). We also found that international airline travel influences the timing of influenza mortality (*r* ^2^ = 0.59; *p =* 0.016). The flight ban in the US after the terrorist attack on September 11, 2001, and the subsequent depression of the air travel market, provided a natural experiment for the evaluation of flight restrictions; the decrease in air travel was associated with a delayed and prolonged influenza season.

**Conclusions:**

We provide the first empirical evidence for the role of airline travel in long-range dissemination of influenza. Our results suggest an important influence of international air travel on the timing of influenza introduction, as well as an influence of domestic air travel on the rate of inter-regional influenza spread in the US. Pandemic preparedness strategies should account for a possible benefit of airline travel restrictions on influenza spread.

## Abstract 2

Influenza timing and spread in the US from 1996 to 2005 was influenced by the volume of domestic and international air travel. The flight ban after September 11, 2001, was associated with a delayed and prolonged influenza season.

## Editors' Summary

**Background.:**

In both the northern and southern hemispheres, influenza epidemics occur annually during the winter “flu season.” Although the disease maps out a remarkably similar pattern in most years, little is known about the specific mechanisms by which geographic spread occurs. Given the perennial possibility of influenza global epidemics (pandemics) such as occurred in 1918, 1957, and 1969, as well as the more recent, localized outbreaks of avian influenza (“bird flu”) in which a high proportion of affected people have died, we need to understand how influenza spreads in order to limit the destructive impact of future pandemics.

**Why Was This Study Done?:**

In theory, airline travel might be expected to play a role in the spread of influenza across large distances. If so, reducing or restricting air travel might be an appropriate public health intervention in the early stages of an influenza pandemic. This study was performed to identify specific effects of air travel on the annual spread of influenza in the United States.

**What Did the Researchers Do and Find?:**

The researchers analyzed weekly government records on deaths from influenza and pneumonia in cities from nine regions of the US during the nine influenza seasons between 1996 and 2005. For each year, they determined the time it took for the epidemic to spread across the US and the date of the national peak in influenza deaths. They then used government estimates of passenger air travel to explore any connection with the timing of the annual flu epidemics.

The analysis found that the usual time for an influenza epidemic to reach peak levels across the US was approximately two weeks, and that the national peak date fell within two days of the average date, February 17, in five of the nine seasons. In general, influenza was found to spread more slowly during years when the number of domestic air travelers, particularly during November, was lower. Also, the peak of the influenza season was found to come later during years when the number of international air travelers, particularly in September, was lower. These results, based on reported deaths from pneumonia or influenza, were corroborated using data from an influenza virus surveillance program, and could not be explained by variations in winter temperatures or by different types of influenza virus circulating in different years.

Of note, the peak date of the US influenza season following September 11, 2001, was delayed by 13 days to March 2, consistent with marked reductions in airline travel following the terrorist attack, and then returned to February 17 over the subsequent two influenza seasons as international airline travel returned to its previous levels. In contrast, the investigators found no delay in the 2001–2002 influenza season in France, where flight restrictions were not imposed.

**What Do These Findings Mean?:**

While this study does not demonstrate that travel restriction would be effective in altering the course of a flu pandemic, it does provides evidence that air travel plays a significant role in the annual spread of influenza in the United States. Although other factors, related or unrelated to the decrease in air travel after September 11, may have affected the course of the 2001–2002 influenza season, the general findings across several years suggest that air travel affects both the peak date and the rate of spread of influenza. These findings merit consideration in the process of preparing for the next influenza pandemic.

**Additional Information.:**

Please access these Web sites via the online version of this summary at http://dx.doi.org/10.1371/journal.pmed.0030401.

- World Health Organization: influenza pandemic preparedness page
- US Department of Health and Human Services: avian and pandemic flu information site
- Wikipedia page on influenza pandemic (note: Wikipedia is a free Internet encyclopedia that anyone can edit)

## Introduction

The influence of air travel on the geographic spread of influenza has been the subject of a number of simulation studies [1–4]. Discrete time SEIR (susceptible–exposed–infectious–recovered) models coupled with air transportation data have been used to explain the global path of influenza epidemics [3] and pandemics [5]. However, there is surprisingly little empirical information on how airline travel influences the spread of influenza through regions, nations, and across the globe. Although recent work suggests high geographical coincidence of time series of influenza mortality at the hemispheric [6] and national scale [7–10], little is known about how epidemics may be connected across large areas. Analyzing spatial–temporal patterns of influenza epidemics represents a critical step toward understanding how population movement contributes to epidemic fluctuations, and will help inform the evaluation of targeted control strategies.

A recent study examined the between-state progression of inter-pandemic influenza in the United States and found a strong relationship with movement of individuals to and from their workplace [10]. Although this local travel may be largely responsible for spread within a region (for example, a state, where travel is dominated by personal vehicular movement), inter-regional influenza spread may be more significantly influenced by long-range airline travel, which comprises almost half of all movement at distances greater than 1,000 miles and the majority of travel at over 2,000 miles [11]. Understanding the role of airline travel in large-scale influenza spread is especially important given the mounting threat of an influenza pandemic [12–14]. The decision of whether travel restrictions should be put into place when a pandemic strain emerges beyond the source is currently under consideration by the World Health Organization [15].

**Figure 1:**
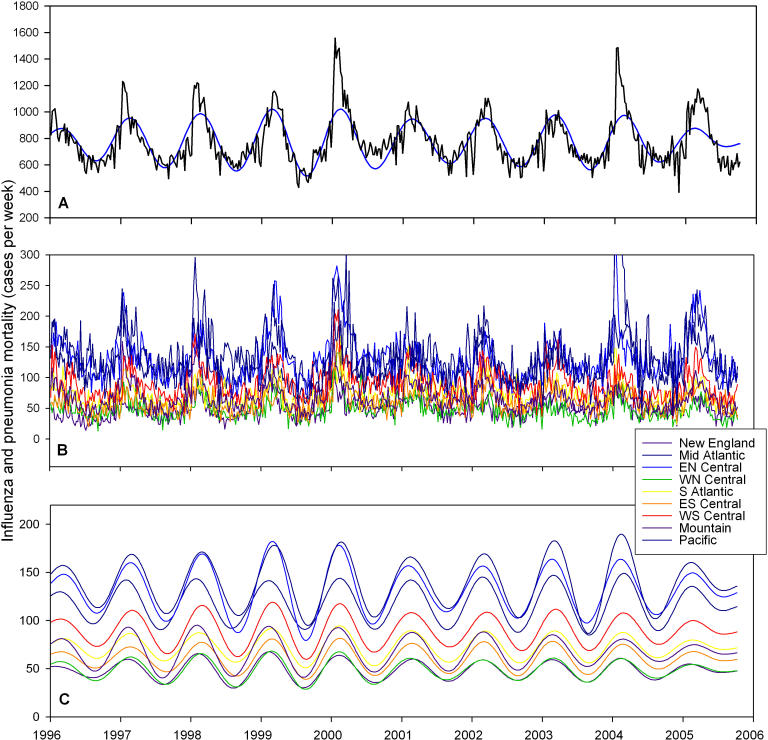
Filtering of Weekly P&I Mortality in the United States (1996–2005) (A) The black line represents the aggregated national data of P&I weekly mortality. The blue line represents the seasonal influenza curve, derived by band-pass filtering the demeaned data (two-pole, two-pass Butterworth, 1/64–1/40 frequency range). For comparison with the raw data, the mean is added after filtering. The filtered time series plus mean accounts for 99.8% of the mortality, indicating that most deaths are from the mean and seasonal variation and not the high-frequency cycles. (B) Lines represent the raw time series data for each of the nine geographic regions of the US. (C) Lines represent the seasonal influenza curves for each of the nine geographic regions of the US, derived by band-pass filtering.

In this study, we characterize the spatial variability in the inter-regional timing of the seasonal component of influenza mortality across the United States and assess its relationship to airline volume. Influenza epidemics peak each year during the winter in the Northern and Southern Hemisphere; thus, epidemics at a particular geographic location typically display strong seasonal cycles (Figure 1). Here, we apply signal processing methods to disease surveillance data to resolve spatial–temporal patterns in the seasonal cycle of inter-regional influenza spread across the US. Based on these patterns, we examine how international and domestic airline travel may influence both the introduction of new viral strains and their spread.

## Methods

### Spatial–Temporal Patterns of Influenza Mortality

**Figure 2:**
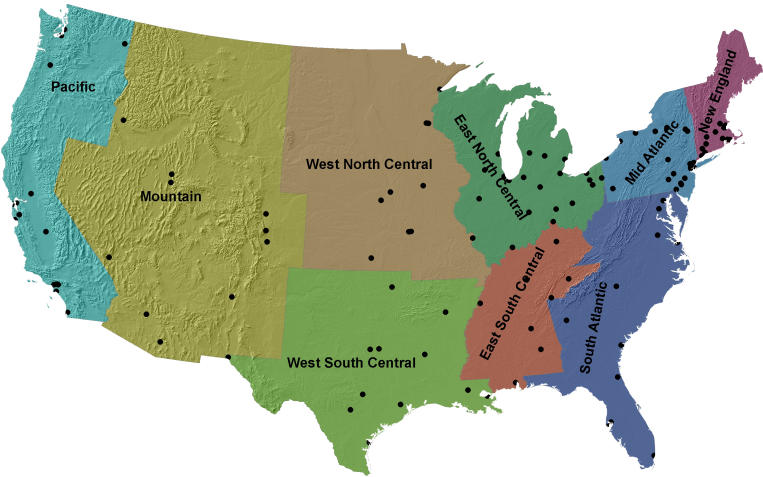
Major Geographic Regions of the United States The sentinel cities that report mortality due to P&I used in the Centers for Disease Control and Prevention 122 Cities Mortality Reporting System are displayed (black dots). Because the strength of the seasonal influenza cycle is weak for cities with small case counts and because some city data contain missing data points, we aggregated the raw city-level data to obtain composite waveforms by major geographic region, the aerial unit of analysis for this study.

Data on weekly mortality from pneumonia and influenza (P&I) were obtained from the Centers for Disease Control and Prevention 121 Cities Mortality Reporting System (http://www.cdc.gov/EPO/DPHSI/121hist.htm) for nine influenza seasons, from 1996–1997 to 2004–2005, representing 396,506 deaths [16]. Because the strength of the seasonal cycle is weak for cities with small case counts and because some city data contain missing data points, we stacked the raw city-level data to obtain composite waveforms for each of the nine major geographic regions of the United States, as defined by the Centers for Disease Control and Prevention (Figure 2). If the noise in the city data is random, such stacking should improve the observation of any coherent regional signal.

For each region, we characterized the seasonality of P&I mortality by filtering. We use band-pass filtering to focus on the seasonality of influenza mortality (Box 1). Specifically, to isolate the seasonal (annual) cycles of influenza mortality we band-pass filtered each of the regional time series using a two-pole, two-pass (zero phase) Butterworth filter with low and high cutoff periods of 40 and 62 wk. Prior to filtering, time series were demeaned, and tapered at the ends to zero to reduce edge effects.

**Box 1.** Time series analysis is a well known method for revealing time-dependent phenomena that are not necessarily apparent in raw data. Because of the strong seasonality of influenza mortality, we used band-pass filtering to isolate the patterns around the yearly (seasonal) signal of influenza. Such filtering essentially smoothes over and removes variations at short time scales (such as daily changes) and long time scales (such as biennial) to isolate the coherent seasonal patterns (Figure 1). Essentially, we extract a range of seasonal frequencies of interest from the time series while rejecting (attenuating) frequencies outside that range. Our filtering approach reflects the fact that, from a time series perspective, the seasonality of influenza mortality is nearly stationary for these data in that the peaking always occurs at similar times in the winter months. This is in contrast to other infectious diseases such as measles and dengue, where strong non-stationary signals are observed and more sophisticated analysis methods, such as wavelets and empirical mode decomposition, are appropriate.

For each influenza year, coincidence in the timing of seasonal influenza mortality across geographic regions was estimated from the phase shift with a national seasonal curve, derived by summing of all city data and filtering. We used spline resampling to achieve daily resolution. We divided the filtered data into subsets by influenza year (week 40 of one year to week 39 of the following year). We then performed cross-correlation with the national time series for each possible comparison (nine regions times 9 y) to estimate phase shifts (lag or lead times), considering a shift range of ±20 wk. The phase shift with the maximum cross-correlation served as an estimate of the relative timing of the seasonal influenza curve in a given region and a given year. We also estimated the peak date of the seasonal national curve for each year. For each year, the time required for an influenza wave to spread across the US was estimated by the variability in the seasonal phase shifts for the nine regions. We used the variation in the phase shifts from the national curve for each year as estimated by the 99% confidence interval to approximate the time to transnational spread.

### Effect of Airline Volume on Inter-Regional Influenza Spread and Peak

We modeled changes in the rate of inter-regional spread of seasonal influenza mortality as a response to yearly fluctuations in domestic airline volume. Monthly estimates of passengers on domestic flights were obtained for November to January of each influenza season [17]. This range was selected because influenza activity begins to increase in November [18], and viral isolate collections by the World Health Organization and National Respiratory and Enteric Virus Surveillance System (WHO/NREVSS) collaborating laboratories show that all regions have influenza activity as of January each year.

We also investigated the effect of international airline travel on the absolute timing of the seasonal peak of national influenza mortality. Monthly estimates of passengers on international flights were obtained for September to November of each influenza season [17]. We selected this range as the most likely time window in which new viral strains would be introduced each influenza season. We used the peak date from the filtered national curve as the indicator of the absolute timing of influenza mortality for a given year.

We fit stepwise regression models to both time to transnational spread and peak timing using domestic and international airline travel volume, respectively. A normal response distribution was used in both cases after analysis of the residuals and statistical tests of normality, including the Kolmogorov-Smirnov and Shapiro-Wilk tests. For each model, we evaluated covariates in a stepwise fashion. Our model for inter-regional influenza spread included overall domestic airline volume for October, November, and December as separate covariates. Our model for influenza peak included overall international airline volume for September, October, and November as separate covariates. In each case, we included a linear trend term to account for the potential effect of improved city reporting over time. We also assessed significance after applying a Bonferroni correction to adjust for the effect of testing across multiple months.

In order to investigate other possible contributing factors, we also included the effect of winter severity and dominant strain in our stepwise regression model [19]. First, we collected climate data to account for the effect of winter temperature. Past studies have shown that colder conditions promote human indoor crowding and thus increased virus transmission and possibly a faster course of virus spread [20,21]. We obtained data from the National Climatic Data Center on national average winter temperature (December–February) and included this as a term in our model [22]. In addition, we examined the effects of the temperature of individual winter months as covariates. We also calculated the minimum mean temperature for a winter period and examined its effect on inter-regional influenza spread and peak. In any given season, a number of strains of varying virulence and spatial distribution can be circulating. Previous research has shown that the dominant circulating subtype is associated with the impact and rate of spread of influenza epidemics [7,10,19]. Thus, strain variation could have an effect on our measures. In order to account for this factor, we included the dominant subtype (A/H3N2 or A/H1N1) as a categorical variable in our model. Finally, previous work has shown that, at the state level, time to transnational spread is influenced by the first state to be affected [10]. Therefore, to account for this potential confounding, we also included the first region with activity identified with the phase shift analysis as a covariate in the model. Model fitting was performed in SAS version 9.0 for Windows (SAS Institute, Cary, North Carolina, United States).

### Model Validation

The P&I mortality data have limitations, including spatial and temporal variation in voluntary reporting and uncertainty about the proportion of deaths attributable to epidemic influenza. Therefore, we validated mortality patterns with viral surveillance data from the WHO/NREVSS collaborating laboratories from 1997–2005. These viral data provide time series of the percentage of positive influenza specimens for an influenza season (from week 40 of one year to week 20 of the following year). High-quality data were available at the national scale for the eight influenza seasons from 1997–1998 to 2004–2005, and at the regional scale for the six influenza seasons from 1999–2000 to 2004–2005. For each season, we calculated the national peak dates of viral activity. Additionally, we calculated the yearly time to transnational spread based on peak week of regional viral activity available from 1999–2005.

In order to establish the causal link between flight reductions in the US after the terrorist attack on September 11, 2001, and a delayed epidemic peak, we examined whether a similar delay occurred in Europe, where flight restrictions were not imposed. We obtained weekly influenza-like illness data for France from 1996–2005 from the French Sentinel Network. This voluntary surveillance system, active since 1984, collects reports from general practitioners across France [23]. We estimated the peak week of yearly influenza epidemics by two methods. First, we estimate peak week by the raw time series and taking the week of highest incidence. Second, we applied our filtering approach described above to estimate peak influenza activity from the smoothed time series. This smoothing could potentially provide a more robust estimate of peak date.

## Results

### Spatial–Temporal Patterns of Influenza Mortality

Our filtering approach reflects the fact that the seasonality is nearly stationary. Spectral analyses of national influenza mortality data confirm that the yearly (~52 wk) Fourier component is the dominant period and that a seasonal time series plus mean can explain 99.8% of the national mortality. Our analyses do not examine the high-frequency epidemic peaks, which were found to be extremely noisy and poorly defined for many influenza seasons (for example, the 2000–2001 and 2002–2003 seasons) and may be more influenced by imperfect reporting (Figure 1B). In contrast, the peaks in the seasonal curves are coherent and well-behaved across the nine regions, and therefore should be reliable proxies of peak influenza mortality (Figure 1C).

**Figure 3:**
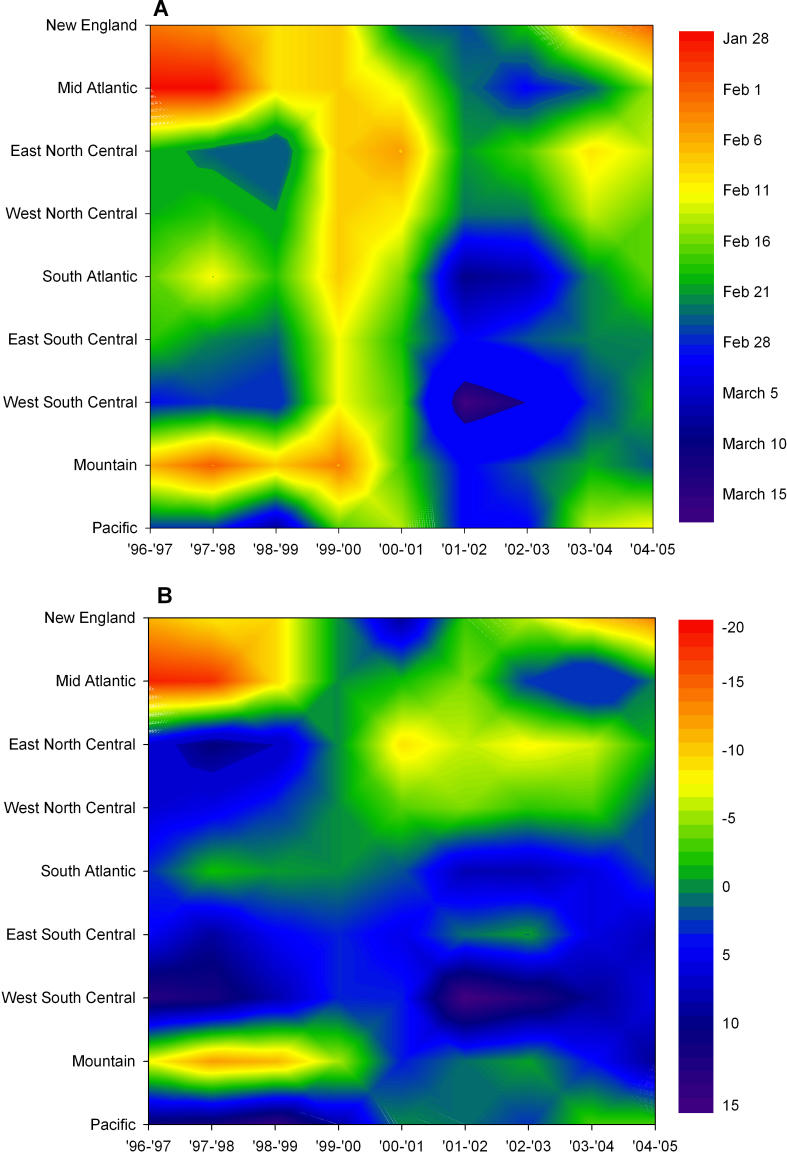
Timing of Influenza Illness across the Nine Major Geographic Regions of the United States (1996–2005) For each influenza year, phase shifts are calculated as the maximum value from cross-correlation of the band-pass filtered weekly P&I mortality data. (A) Contour plot of raw phase shifts between regions for each season, which displays shifts in the absolute timing of influenza mortality peaks from year to year. The plot shows the shifts in the yearly phase, with the 1999–2000 season exhibiting an overall earlier peak and the 2001–2002 season (following September 11, 2001) exhibiting an overall later peak across all regions. (B) Contour plot of demeaned phase shifts, which displays typical regional patterns and relative time to transnational spread. For each season, demeaned phase shifts were calculated by subtracting the mean peak date. The plot reveals increased variation in phase shifts (time to transnational spread) during the earliest influenza seasons, 1996–1997 and 1997–1998, as well as the increased variation during the 2001–2002 influenza season.

Although the sequence of infection varied among regions from year to year, certain spatial–temporal patterns emerged in the seasonal component of P&I mortality (Figure 3). The yearly component of influenza takes approximately 2 wk on average to peak over all US regions. The time to transnational spread decreased from 24–26 d during the 1996–1997 and 1997–1998 influenza seasons to 8–11 d during 1999–2000 and 2000–2001 seasons. The 2001–2002 influenza season (following September 11, 2001) manifested an increase, with a time to transnational spread of 16 d, 68% longer than for the previous two seasons. In the subsequent influenza seasons, there was only a slight decrease in rate of inter-regional spread, to an average of 15 d, and there was not a return to the rate of before September 11, 2001.

We found a significant effect of influenza season on the phasing of the overall national curve (analysis of variance, *f*(1,8) = 3.931; *p =* 0.001). We found that the national peak date for seasonal influenza mortality was stable for five of the nine seasons, occurring within 2 d of February 17. The influenza season following September 11, 2001, had a markedly delayed peak, on March 2, 2002, 13 d later than average. The subsequent influenza seasons, 2002–2003, 2003–2004, and 2004–2005 progressively returned to baseline, with peaks on February 29, 19, and 17, respectively.

### Effect of Airline Volume on Influenza Inter-Regional Spread and Peak

**Figure 4:**
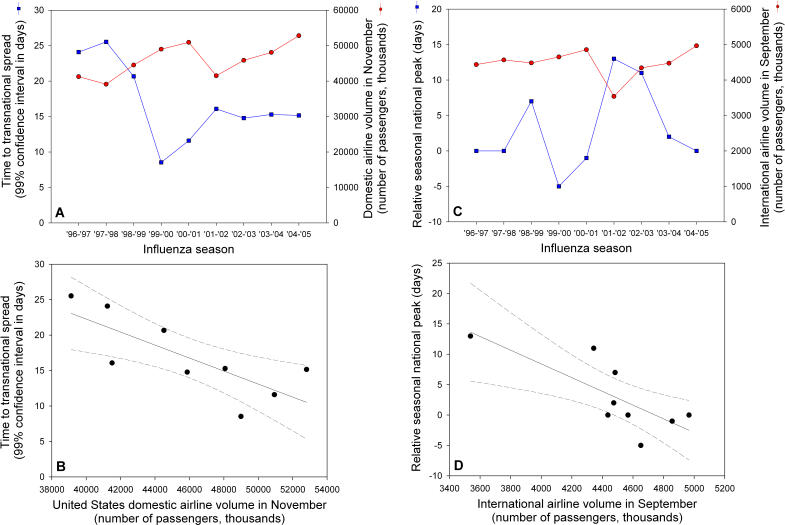
Influence of United States Airline Volume on Influenza Spread and the Timing of Yearly Transmission (A) November domestic air travel volume (red line) is estimated by the total number of passengers on domestic flights. Duration to transnational spread of influenza (blue line) is estimated as the 99% confidence intervals for differences between the estimated seasonal curves of influenza mortality for each of nine major geographic regions of the United States. (B) The association between domestic airline travel in November and transnational spread is displayed. The numbers of traveling domestic passengers in November significantly predicts transnational influenza spread (*f* = 10.6; *r* ^2^ = 0.60; slope = −0.94 days/million passengers; *p =* 0.014). (C) September international air travel volume (red line) is estimated by the total number of passengers on international flights. The timing of seasonal national influenza mortality (blue line) is estimated as the peak date of influenza mortality from the filtered national curve. The timing displayed is relative to the average date of February 17. (D) The association between international airline travel in September and the timing of the US influenza peak is displayed. The numbers of traveling international passengers in September significantly predicts the timing of seasonal influenza mortality (*f* = 10.0; *r* ^2^ = 0.59; slope = −11.3 days/million passengers; *p =* 0.016).

We found that changes in the rate of spread and timing of seasonal influenza mortality were correlated with yearly fluctuations in monthly airline volume (Figure 4A and 4B). An inverse correlation was found between time to transnational spread of influenza and the number of traveling domestic passengers during the November to January period (Pearson correlation, *R* = −0.69; *p =* 0.021). Though each of the three months reveal an inverse relationship, we found that domestic airline volume in November was the single significant predictor of influenza spread (*r* ^2^ = 0.60; *p =* 0.014) (Figure 4). This relationship was especially evident for the 1997–1998 season, in which there was both the lowest airline volume (39 million passengers) and the slowest spread (26 d).

A strong inverse correlation was found between the timing of an influenza season and the numbers of traveling international passengers between September and November (Pearson correlation, *R* = −0.66; *p =* 0.027) (Figure 4). In this case, although all three months showed an inverse relationship, international travel in September was the single month to significantly predict the seasonal national peak (*r* ^2^ = 0.59; *p =* 0.016). This is especially evident during 2001–2002, when international flight volume decreased by 27%, from 4.9 million international passengers to 3.5 million, and peak influenza mortality was delayed by 2 wk. Furthermore, we also found a delayed peak during the 2002–2003 season, when international airline travel was down by 10% because of the residual effect of the events of September 11, 2001, on travel behavior. A continued trend back to baseline peak was found during the 2003–2004 and 2004–2005 seasons as international airline activity resumed its levels of before September 11, 2001. Relationships for both influenza spread and peak were still significant after application of the Bonferroni correction to account for multiple testing of individual months (alpha = 0.017).

We did not find a significant relationship between climate and inter-regional influenza spread. Although we did find a 2001–2002 warm temperature spike and a positive relationship between hot temperatures and late peaking, this relationship was not significant and dropped out of the stepwise regression model. Indeed, as the 2001–2002 season contained the second warmest November–February period on record, environmental conditions may have contributed to the late national peaking of influenza in that season. However, the 1999–2000 season was the warmest November–February period since 1895 and yet had an earlier than average national influenza peak**.** We also considered winter months separately, as well as overall minimum mean winter temperature, but none were significant predictors. In addition, strain type did not account for any significant amount of the variability in transnational spread or peak time of influenza mortality. Finally, first region affected was also not a significant explanatory variable (see Protocol S1).

### Model Validation

Viral data from the WHO/NREVSS collaborating laboratories were used to validate seasonal patterns obtained from the filtered mortality data. We found that peaks in the seasonal mortality data occurred about a month after those in the viral data (mean delay = 30.8 d; 95% confidence interval: 9.1–52.4 d). The estimated spread and peak of the filtered mortality and viral data were well correlated, with Spearman rank correlations of 0.928 (*p =* 0.004) and 0.695 (*p =* 0.028), respectively. Our validation with the viral data indicates that although the absolute scaling between influenza activity and seasonal mortality differs, the relative ordering of peak dates and time to transnational spread between these two datasets is consistent. Furthermore, analysis of the viral surveillance data confirms the effect of September 11, 2001. We found a significantly longer time to transnational spread and a delayed peak date for the 2001–2002 season. The time to transnational spread was 53 d, 60% longer than the average of 33 d, which is a statistically significant difference (*p* < 0.001). The national viral peak date for the 2001–2002 season was calculated at February 23, significantly later than the average of January 20 across the other seasons (*p =* 0.012).

Unlike in the United States, we did not see a similarly delayed peak of influenza activity during the 2001–2002 season in France, where flight restrictions were not imposed. For estimation based on both the raw and filtered time series, the defined peak during this season was estimated at the fourth week in January, 2002. This peak week was not significantly different than that of the eight other influenza seasons (for the raw time series, mean peak was the fourth week; 95% confidence interval: 0–9 wk; for the filtered time series, mean peak was the third week; 95% confidence interval: 1–5 wk). This result provides further evidence that the delayed 2001–2002 US influenza mortality peak was linked to the flight restrictions following the events of September 11, and the subsequent depressed air travel market.

## Discussion

This study is an empirical analysis of the spatial–temporal pattern of inter-regional influenza spread across the United States and provides evidence for factors that influence it. Whereas previous simulation models have suggested that air travel may play an important role in the spread of annual influenza [1,3], we provide what is to our knowledge the first empirical evidence to confirm the effect airline volume on long-range spread. Our findings suggest that once introduced, new viral strains are likely to spread rapidly across geographic regions. Furthermore, though between-state movement may be driven primarily by workflows [10], our results suggest that inter-regional spread occurs by a different mechanism, where air travel may be an important mode of long-range dissemination of influenza. We find that the effect of airline volume on regional influenza spread is largely based on travel in November. Though influenza activity is highest between January and March, initial regional seeding of infection may occur earlier. Our results suggest that for a non-pandemic year, travel during the Thanksgiving holiday may be central to the yearly national spread of influenza in the US. Similarly, we found that international airline travel influences the absolute timing of seasonal influenza mortality.

The flight ban in the US after the terrorist attack of September 11, 2001, and the subsequent depression of the air travel market provided a natural experiment for the evaluation of the effect of flight restrictions on disease spread. The importance of airline activity was highlighted by the delayed peak of influenza in 2001–2002 following the period of reduced flying activity. This finding is further validated by the absence of a similar delay in influenza activity in France, where flight restrictions were not imposed. Our model suggests that September may be the critical month for entry of new influenza strains into the US from foreign countries, earlier than the established start of the US influenza season in October/November. Although seasonal influenza activity usually begins to increase as early as October or November, current laboratory surveillance by the WHO/NREVSS collaborating laboratories consistently collects viral isolates in its first week of testing (week 40; first week of October). Over the last eight influenza years (1997–1998 to 2004–2005), 0.62% (standard deviation = 0.59%) of specimens on average test positive for influenza in the first week of October, indicating that the introduction of new viral strains has already occurred in September. Indeed, new antigenically distinct strains result from a continuous evolutionary process of small changes in influenza surface antigens and are not limited to a given location or time period [24], and therefore international travel in September can surely not be the only mechanism of strain introduction.

While our study suggests that airline passenger volume explains about 60% of the inter-annual variation in inter-regional influenza spread and peak, there is still an unexplained component. The timing of seasonal influenza mortality could reflect the additional influences of climatic conditions [19,25] rather than solely the introduction of new strains into a susceptible population by airline travel. However, we find that monthly national temperatures were not a significant predictor in our models. Another issue is that strain variation could have an effect on our measures. Our models included a term for dominant subtype, which was not found to be significant***.*** Recent studies have shown that influenza spreads more efficiently during seasons dominated by subtype A/H3N2 than when A/H1N1 or B dominate [7,9,19,26]. For instance, the 1981–1982 and 1990–1991 seasons, which were dominated by influenza B, were substantially less synchronous than other seasons of the 1980s and 1990s [10]. Interestingly, the 2001–2002 season (after September 11, 2001), where we found delayed spread, was dominated by A/H3N2 circulation. In this case, lack of synchrony cannot be explained by dominant subtype, which suggests that other factors (including reduced airline travel) may have been responsible. In fact, our study period from 1996 to 2005 represents the longest stretch of A/H3N2 season in over 30 y (seven out of nine seasons were dominated by A/H3N2), essentially controlling for the effect of subtype. Yearly changes in public health intervention strategies, such as vaccination campaigns, and vaccine efficacy may also affect patterns of spread. Improved vaccination coverage or strain match in a given season would decrease the rate of disease transmission and thus also slow the rate of spatial spread [27,28]; such a scenario could have potentially caused the delay that occurred in 2001–2002. Studies of influenza mortality based on US multiple cause-of-death files [29] provide longer and more comprehensive time series, and enable a more detailed analysis of these multiple effects. However, yearly data become available only after 3 y, and therefore cannot be used for the current analysis. In contrast, data from the 121 Cities Mortality Reporting System provide a more current time series of influenza mortality that is available for examining the recent fluctuations in human travel, including evaluating the effect of the 2001–2002 flight reduction on influenza spread.

Our study does, however, have certain limitations that are inherent in the use of mortality data from the 121 Cities Mortality Reporting System. One limitation is associated with the voluntary design of the system. There is variability in time of filing of death reports from week to week because of changes in volunteer staff and insufficient staff to keep up with reporting during the peak of the influenza season. We observed that reporting quality varies with both time and city, as evidenced by the presence of gaps (weeks with no data) and anomalous behavior in some of the city time series. We therefore stacked the raw city data according to major geographic regions. This stacking enabled us to extract coherent regional seasonal signal from the P&I data. These results lead us to believe that the noise in the city data was random and that there were no systematic biases that would account for our findings. Furthermore, P&I mortality has been validated as a good relative proxy for the severity of influenza epidemics [30]. Thus, the use of these P&I data to estimate relative seasonal curves of influenza mortality should be appropriate as well.

We used influenza mortality time series data, which may not correspond precisely to influenza activity. P&I mortality reflects a somewhat uncertain mixture of deaths from influenza and other respiratory diseases, and the proportion of influenza deaths may vary with time. Furthermore, although influenza strikes all age groups, non-pandemic influenza mortality predominantly affects the elderly, and older age groups typically peak later, while young children peak earlier [31]. There may also be other factors related to the biology of disease progression and associated complications that cause timing differences between influenza morbidity and mortality. However, our analyses show that influenza mortality patterns correspond with trends in virological data from laboratory surveillance, which suggests that we have captured a true pattern of influenza timing and spread. Although a more detailed national dataset of confirmed influenza infections and matched strain information would be ideal, our study demonstrates how analyses of imperfect influenza surveillance data can reveal important spatial–temporal trends, providing potentially vital information for disease prevention and control.

The alarming spread of the highly pathogenic avian influenza A (subtype H5N1) in both wild and domestic poultry in Southeast Asia and Europe [32,33], with probable human-to-human transmission [34,35], has intensified the debate over whether border control and travel restrictions could substantially impede the spatial spread of an emerging pandemic strain. Our results suggest that limiting domestic airline volume would have a measurable impact on the rate of spread of an influenza pandemic, and particularly on spread across regions. Because influenza pandemics have shown unusual spatial and demographic patterns as well as higher basic reproductive number due to lack of immunity, the relationship between air travel volume and domestic influenza spread may nonetheless be different in a pandemic scenario [36,37]. However, our finding that international travel influences the timing of epidemic influenza should apply directly to a pandemic scenario, where the objective will be to reduce the probability of strain introduction.

Recent individual-based simulation models of pandemic influenza transmission have attempted to model the effectiveness of social distance measures, including travel restrictions [38,39]. While eliminating travel in and out of affected areas along with imposing border restrictions may provide some relief by delaying spread by up to 8 wk [38], drastic reductions in domestic travel are required to have much impact internally [38,40]. Although these simulation studies have found that these strategies may not have a significant impact on the course of a pandemic, the models lack parameterization of the underlying relationship between air travel and influenza spread based on experimental data. Future work using simulation will benefit greatly from parameter estimates based on empirical findings such as those presented here.

Although the mechanisms driving the seasonality of influenza epidemics are still not well understood, our findings do suggest that fluctuations in airline travel have an impact on large-scale spread of influenza. At the regional level, our results suggest an important influence of international air travel on influenza timing as well as an influence of domestic air travel on influenza spread in the US. However, for the global influenza pandemic widely believed to be inevitable [41], the efficacy of travel advisories, flight restrictions, or even complete flight bans as a control measure is still uncertain. Though our results suggest a possible benefit of airline travel restrictions, without early detection and immediate action, such measures may be ineffective at stemming the spread or mitigating the impact of an oncoming pandemic [42]. Furthermore, even with a significant travel ban, the rapid rate of influenza spread might still outpace the capability to manufacture and distribute large amounts of vaccine matched to the new variant [43]. Policy-makers will also need to consider and balance the social, constitutional, legal, economic, and logistic consequences of such quarantine measures [44,45].

## Supporting Information

Protocol S1 Additional Methods and Results (844 KB DOC)

## Abbreviations

P&I: pneumonia and influenza
WHO/NREVSS: World Health Organization and National Respiratory and Enteric Virus Surveillance System
