# Supplementary material for: Empirical Evidence for the Effect of Airline Travel on Inter-Regional Influenza Spread in the United States
Source: PLoS Med. 2006 Sep 12;3(10):e401. doi: 10.1371/journal.pmed.0030401 (PMC1564183; doi:10.1371/journal.pmed.0030401)
Supplement: Supplementary file 1 [file pmed.0030401.sd001.doc]

**Supporting Online Material – Protocol S1**

**Data**

Influenza mortality is monitored through weekly reports filed by the vitals statistics offices of 121 cities to the CDC 121 Cities Mortality Reporting System and published weekly in Table III of the Morbidity and Mortality Weekly Report (13). The 121 Cities Mortality Reporting System is a volunteer system where the participating cities use their own systems to count the P&I deaths and report data to the CDC within 2-3 weeks from the date of death. The CDC reports contain the total number of death certificates reported by each city and the number of those for which pneumonia or influenza (P&I) was listed as a contributing cause of death. A death is reported by the place of its occurrence and by the week that the death certificate was filed. The system does not provide incidence rates, as deaths are reported to CDC by place of occurrence, and not by place of residence. For non-pandemic influenza years, it has been estimated that most (>80%-90%) influenza-associated deaths occur among persons aged ≥ 65 years (27). Details of these data including the list of participating cities can be found at: [www.cdc.gov/epo/dphsi/121hist.htm](../www.cdc.gov/epo/dphsi/121hist.htm).

Time series of mortality due to pneumonia and influenza were created for each season from early season at the beginning of October (week 40) until late September of the following year (week 39). Mortality data were available beginning January1^st^, 1996 and were grouped by the nine major geographic regions of United States; these include New England, Mid Atlantic, East North Central, West North Central, South Atlantic, East South Central, West South Central, Mountain and Pacific regions.

**Signal Processing**

Time series analyses are well known methods for revealing phenomenon that are not necessarily apparent in raw data. Because of the strong seasonality of influenza mortality, we use band-pass filtering to focus on the patterns around the seasonal (52 week) signal. Our simple filtering approach reflects the fact that, from a time series perspective, the seasonality of influenza mortality is nearly stationary. This is opposed to the diseases of measles and dengue, where strong nonstationary signals are observed and more sophisticated analysis methods, such as wavelets and empirical mode decomposition, are appropriate. We also find that spectral analyses of national influenza mortality data confirms the yearly (~52 week) Fourier component is the dominant period in the data and we note that a 40-62 week band pass filtered time series plus mean can explain 99.8% of the national mortality (Figure 1a).

Our analyses do not examine the pattern of high frequency peaks in the data, such as high frequency outbreaks or the patterns of excess deaths above a seasonal baseline. Using a high pass filtered approach, we have found that the distribution of high frequency peaks is indeed quite complicated (Figure 1). For example, influenza outbreaks can occur any time of the year (though are most common at the winter peaks) and for some influenza seasons the winter high frequency peaks are extremely noisy and poorly defined, such as the 2000-2001 and 2002-2003 seasons (Figure 1). These high frequency patterns, as well as the lower frequency interannual variations, likely reflect additional interesting dynamics of influenza that we do not explore here. In comparison, the band-pass filtered seasonal data produced an extraordinarily well behaved signal that explains the broad patterns of P&I mortality. For this reason, we chose to focus on modeling the overall seasonality of influenza mortality.

For the analyses, prior to filtering, time series were demeaned and tapered at the ends to zero to reduce edge effects and prevent the more poorly constrained results at the ends of the time series from influencing the results. Then, to isolate the seasonal (annual) cycles of influenza mortality we band-pass filtered each of the regional time series using a two-pole, two-pass (zero phase) Butterworth filter with low and high cutoff periods of 40 and 64 weeks. The 40-64 interval (52 +/- 12 weeks) allows sequential yearly shifts of up to +/- 12 weeks in the peaking of the seasonal mortality. Our actual results yield significantly smaller shifts of only +/- 3 weeks (Figure S1), demonstrating how the chosen range for filtering is more than adequate to capture the actual shifts in the data. After the seasonal cycles have been defined, phase shifts are estimated between each of the data sets by cross-correlation. These estimates are measures of the extent to which each frequency component of one series leads the other (Table S1).

**Regression Models**

We fit linear models to both time to transnational spread and peak mortality data using airline domestic and international airline travel volume respectively. A normal response distribution was considered in both cases after analysis of the residuals and the statistical tests of normality, including the Kolmogorov-Smironov and Shapiro-Wilk tests.

For each model, we evaluated covariates in a stepwise fashion. Our model for influenza spread initially included overall domestic airline volume for November, December and January as separate covariates as well as a linear trend terms to account for better reporting with time. We find that airline volume in November was the single significant predictor of influenza spread with a slope of -0.94 days/ million domestic passengers [f=10.6; r^2^=0.60; P=0.014]. Domestic airline volume in October [β=-0.75 days/million domestic passengers; f=4.7; r^2^=0.40; P=0.067] and December [β=-0.71 days/million domestic passengers; f=2.7; r^2^=0.28; P=0.14] were not significant predictors of influenza spread.

Our model for influenza peak initially included overall international airline volume for September, October and November as separate covariates as well as a linear trend terms to account for the possible effect of better reporting with time. In this case, air travel in September was the sole predictor of the peak time of seasonal influenza mortality with a slope of -11.3 days / million international passengers [f=10.0; r^2^=0.59; P=0.016]. International airline volume in October [β=-7.4 days/million international passengers; f=5.0; r^2^=0.42; P=0.060] and December [β=-1.0 days/million international passengers; f=0.061; r^2^=0.0087; P=0.81] were not significant predictors of influenza peak. We also account for the effects of winter temperature, dominant subtype and first region affected as potential confounders to these relationship. (table S2). Model fitting was performed in SAS v. 9.0 for Windows (The SAS Institute Inc., Cary, NC).

**Table S1. Results of cross-correlation of the filtered regional datasets of influenza and pneumonia mortality.** The average temporal phase shifts from the filtered national dataset are displayed. Phase shifts are calculated by the maximum value from cross-correlation of the band pass filtered weekly pneumonia and influenza mortality data. The peak dates of the filtered national seasonal curve are also displayed.

|  | Phase shift from national time series (days) | | | | | | | | |
| --- | --- | --- | --- | --- | --- | --- | --- | --- | --- |
|  | 96-97 | 97-98 | ’98-‘99 | ’99-‘00 | ’00-‘01 | ’01-‘02 | ’02-‘03 | ’03-‘04 | ’04-‘05 |
| New England | -13 | -11 | -13 | -2 | 8 | -4 | -7 | -11 | -14 |
| Mid Atlantic | -20 | -20 | -13 | -2 | -2 | -6 | 1 | 7 | 0 |
| EN Central | 5 | 8 | 5 | -2 | -9 | -7 | -4 | -7 | -2 |
| WN Central | 5 | 3 | 0 | -2 | -4 | -6 | -9 | -4 | 1 |
| South Atlantic | 1 | -4 | -4 | -2 | 1 | 7 | 7 | 5 | 1 |
| ES Central | 3 | 7 | 1 | 1 | 5 | 0 | -2 | 5 | 7 |
| WS Central | 12 | 10 | 5 | 1 | 3 | 15 | 12 | 8 | 5 |
| Mountain | -9 | -14 | -14 | -7 | 3 | 0 | -2 | 3 | 8 |
| Pacific | 10 | 10 | 12 | 7 | 0 | 0 | 1 | -4 | -4 |
|  | | | | | | | | | |
| Time to Transnational Spread  (99% CI, days) | 24.4 | 25.5 | 20.8 | 8.4 | 11.4 | 16.0 | 14.8 | 15.3 | 14.9 |
| National Peak Date | Feb 17 | Feb 17 | Feb 24 | Feb 12 | Feb 16 | Mar 2 | Feb 28 | Feb 19 | Feb 17 |

**Table S2. Results of regression models for the effect of influenza spread and influenza peak.** Changes in the rate of spread and peak of seasonal influenza mortality were modeled as a response to yearly fluctuations in domestic and international airline volume, respectively, by linear regression. The effects of potential confounders including, winter temperature, dominant viral subtype and first region to be affected were also added to the models.

| Model Covariate | Proportion of variation in yearly influenza spread explained (R^2^) | Model Covariate | Proportion of variation in yearly influenza peak explained (R^2^) |
| --- | --- | --- | --- |
| *Domestic airline volume (Nov. to Jan.)* | 0.47 (P=0.042) | *International airline volume (Sept. to Nov.)* | 0.43 (P=0.051) |
| November Travel | 0.60 (P=0.014) | September Travel | 0.59 (P=0.016) |
| December Travel | 0.28 (P=0.15) | October Travel | 0.42 (P=0.06) |
| January Travel | 0.35 (P=0.091) | November Travel | 0.27 (P=0.15) |
| *Winter temperature (Dec. to Feb.)* | 0.045 (P=0.58) | *Winter temperature (Dec. to Feb.)* | 0.006 (P=0.84) |
| *Dominant viral subtype* | 0.14 (P=0.32) | *Dominant viral subtype* | 0.036 (P=0.63) |
| *First region affected* | 0.071 (P=0.49) | *First region affected* | 0.02 (P=0.90) |
| *November Travel controlling for:*  Winter Temperature  Dominant subtype  First region affected | 0.60 (P=0.029)  0.61 (P=0.035)  0.66 (P=0.019) | *September Travel controlling for:*  Winter Temperature  Dominant subtype  First region affected | 0.60 (P=0.025)  0.69 (P=0.012)  0.59 (P=0.026) |

**Figure S1. Analysis of weekly pneumonia and influenza (P&I) mortality for the nine regions of the United States compared to the summed national data (1996-2005).** In the top plot, the raw data for each region is represented by black lines. The summed national time series is in blue. Filtering of each dataset was performed by band-pass filtering the demeaned, tapered data (two-pole, two-pass Butterworth, 1/64 – 1/40 frequency range). The filtered regional data is in red and the filtered summed national data is in magenta. The bottom plot represents the corresponding phase shift (in days) of the filtered regional data with respect to the filtered national data, from cross-correlation analysis. Phase shifts are calculated by the maximum value of the cross-correlation for each subset influenza season (week 40 to week 39 of the following year). Each of the nine panels represent the nine major geographic regions of the United States: (**A**) New England; (**B**) Mid-Atlantic; (**C**) East North Central; (**D**) West North Central; (**E**) South Atlantic; (**F**) East South Central; (**G**) West South Central; (**H**) Mountain; (**I**) Pacific.

**Figure S1(a).**

**Figure S1(b).**

**Figure S1(c).**

**Figure S1(d).**

**Figure S1(e).**

**Figure S1(f).**

**Figure S1(g).**

**Figure S1(h).**

**Figure S1(i).**
